# Oestrogen receptor status, pathological complete response and prognosis in patients receiving neoadjuvant chemotherapy for early breast cancer

**DOI:** 10.1038/sj.bjc.6602235

**Published:** 2004-11-23

**Authors:** A E Ring, I E Smith, S Ashley, L G Fulford, S R Lakhani

**Affiliations:** 1 Breast Unit, Royal Marsden Hospital, Fulham Road, London SW3 6JJ, UK

**Keywords:** neoadjuvant chemotherapy, oestrogen receptor, pathological response

## Abstract

The aim of this study was to ascertain if oestrogen receptor (ER) status predicts for pathological complete response (pCR) to neoadjuvant chemotherapy in operable breast cancer, and the effects of pCR on survival. Using a single-institution database, 435 patients were identified, who received neoadjuvant chemotherapy for operable breast cancer and were eligible for the analysis. Patients whose tumours were ER negative were more likely to achieve a pCR than patients who were ER positive (21.6 *vs* 8.1%, *P*<0.001). Owing to a strong correlation between ER status and grade, these variables were not shown to be independent predictors of pCR. Overall survival (OS) was better in those patients who achieved a pCR compared to those who did not (5-year OS 91 *vs* 73%; *P*=0.02). This was still the case when only patients with ER-negative tumours were examined (5-year OS 90 *vs* 52%, *P*=0.005), but not in the subset of patients with ER-positive tumours (5-year OS 93 *vs* 79%; *P*=0.3). Therefore, patients with ER-negative tumours were found to be more likely to achieve a pCR to neoadjuvant chemotherapy than those with ER-positive tumours, and pathological response did not have prognostic significance in patients with ER-positive tumours.

Neoadjuvant chemotherapy is increasingly being used in the management of patients with large operable and locally advanced breast cancers. This treatment is administered in the hope that downstaging might avoid mastectomy, as an *in vivo* measure of chemosensitivity, and to enable systemic treatment of occult micrometastatic disease (Smith and Lipton, 2001). Randomised trials comparing pre-operative and post-operative adjuvant chemotherapy in early breast cancer show similar rates of local control and overall survival (OS), but the mastectomy rate is lower with the pre-operative approach (Scholl *et al*, 1994; Powles *et al*, 1995; Fisher *et al*, 1998).

Existing studies show that the patients who benefit the most from neoadjuvant chemotherapy are those who achieve a pathological complete response (pCR) with no residual microscopic tumour. This is relatively uncommon, occurring in only 3–16% of patients (Powles *et al*, 1995; Bonadonna *et al*, 1998; Fisher *et al*, 1998; Buzdar *et al*, 1999; Kuerer *et al*, 1999; van der Hage *et al*, 2001), but it is important because patients achieving a pCR have a substantially improved disease-free survival (DFS) and OS compared to those with pathological evidence of residual cancer (Bonadonna *et al*, 1998; Fisher *et al*, 1998; Kuerer *et al*, 1999). In order to identify those most likely to achieve benefit from neoadjuvant chemotherapy, it would be useful to be able to identify predictors of pCR prior to commencing treatment.

Recent data, published in abstract form, have suggested that negative oestrogen receptor (ER) status may be predictive of pCR in patients receiving neoadjuvant chemotherapy for operable and locally advanced breast cancer (Buzdar *et al*, 2003; Euler and Tulusan, 2003). We have tested this hypothesis in a retrospective analysis of ER status as a variable to predict pCR in the breast and axilla in patients receiving neoadjuvant chemotherapy at the Royal Marsden Hospital.

## PATIENTS AND METHODS

### Patient selection

A sequential and prospectively maintained database was retrospectively searched for patients who received neoadjuvant chemotherapy for primary operable breast carcinoma. Patients with locally advanced disease (defined as inoperable by the Haagensen criteria (Haagensen and Stout, 1943)) or inflammatory breast cancer were excluded from this analysis. Patients were only eligible if the diagnosis had been confirmed histologically (by core biopsy) prior to commencement of chemotherapy, and they had undergone surgery following chemotherapy. Metastatic disease was excluded using chest X-ray, complete blood count, and biochemical liver and bone profiles; further investigations were only carried out if clinically indicated. Patient characteristics including clinical stage, menopausal status, tumour grade, ER status, type of chemotherapy and pathological response in breast and axilla were recorded. Patients who received neoadjuvant chemotherapy between 19 February 1985 and 18 February 2003 were eligible for this analysis. Data available up to 30 September 2003 were used.

### Treatment

Neoadjuvant chemotherapy included: (1) anthracycline-based regimens using epirubicin 60 mg m^−2^ or doxorubicin 60 mg m^−2^; (2) cyclophosphamide (CMF) 100 mg orally on days 1–14, methotrexate 30 mg m^−2^ on days 1 and 8, 5-fluorouracil 1 g m^−2^ on days 1 and 8; and occasionally (3) mitoxantrone-containing regimens (up to 11 mg m^−2^). Six cycles of treatment were planned, given once every 3 weeks, although some of the early infusional regimens involved eight cycles of treatment. Response after each cycle was evaluated by clinical measurement of the two largest diameters, and graded according to standard WHO criteria. All chemotherapy was delivered prior to local therapy, post-operative adjuvant chemotherapy was not given.

Following chemotherapy, all patients included in this analysis underwent surgery (conservative or mastectomy). Up until 1995, patients achieving a clinical complete response had been offered the option of not having surgery. However, this practice was stopped following an audit, which suggested a high local recurrence rate; these patients were excluded from the current analysis and have been reported elsewhere (Ring *et al*, 2003). All patients undergoing breast-conserving surgery were given breast radiotherapy. The range of doses given to the breast was 46–50 Gy, with boosts to tumour bed at 11.1–17.5 Gy. Our policy was also to give radiotherapy to the axilla if surgically untreated (dose range 46–50 Gy), and to the supraclavicular fossa (dose range 46–50 Gy), where axillary node status was positive or unknown. Radiotherapy was also given to patients with involved axillary nodes after mastectomy. Tamoxifen (20 mg) was given to most patients within the context of clinical trials or according to standard practice at the time.

### Follow-up

Patients were reviewed after each cycle of chemotherapy for clinical response. Following surgery or radiotherapy, they were reviewed every 3 months for 2 years, then 6 monthly until 5 years. Thereafter, they were assessed clinically and with mammography annually.

### Immunohistochemical analysis of ER status

Oestrogen receptor status was assessed on the diagnostic core biopsy specimen prior to the commencement of chemotherapy. For 254 of the final 382 patients for whom ER status was available, this was ascertained at the time of initial treatment. For the remainder, paraffin blocks were acquired from the archives and ER status ascertained for the purposes of this analysis.

All ER assays were conducted by a laboratory, which participated in the relevant UK National External Quality Assessment Scheme throughout this period. Until 1992 ER was measured by multiple-point ligand-binding/dextran-coated charcoal assay (DCC), with values being obtained by Scatchard plot analysis. Between 1993 and December 1994, ER was measured by enzyme immunoassay (EIA) using kits (Abbott Diagnostics, Chicago, IL, USA). From 1995 onwards, an immunocytochemical assay was used with either the DAKO 1D5 or Novocastra 6F11 antibodies (Dako, Cambridge, UK and Novocastra Laboratories Ltd, Newcastle upon Tyne, UK). Antigen retrieval, incubation with primary and secondary antibodies, and development using streptavidin ABC-horseradish peroxidase were performed as described previously (Detre *et al*, 1999). From March 2003, this process was performed in a semiautomated manner using the Ventana Benchmark™ system (Ventana Medical Sytems, Illkirch, France). Progesterone receptor and HER2 status were only available for a few patients and were therefore not included in this analysis.

In both DCC and EIA assays, values of ⩾10 fmol mg^−1^ protein were considered as positive. When slides were analysed by immunohistochemistry, a score was assigned which estimated the proportion of positive-staining tumour cells (0, 1, 10 or 100%), and the intensity of staining (0, none; 1, weak; 2, intermediate; 3, strong). Tumours were defined as positive if ER staining of any intensity was seen in 10% or more of cells.

### Histological examination of tissue

A pCR was defined as no residual invasive disease in the breast or regional lymph nodes. Where there was only residual DCIS, this was included in the pCR category.

### Statistical methods

The influence of baseline characteristics on the likelihood of achieving a pCR was tested in a univariate analysis by means of the *χ*^2^ test or Fisher's exact test. The independent significance of these variables was assessed in a multivariate logistic regression analysis using a step-up procedure. The odds ratios of a pCR were calculated from the final model. Overall survival and recurrence-free survival were measured from the date of first treatment until death or recurrence, respectively; deaths without recurrence were censored in the recurrence-free survival analysis. Survival curves were calculated by the Kaplan–Meier method and differences were assessed by the log-rank statistic (Kaplan and Meier, 1958; Peto *et al*, 1977).

## RESULTS

### Pathological complete response in the breast

Between 19 February 1985 and 18 February 2003, 439 patients were recorded as having surgery following neo-adjuvant chemotherapy for primary operable breast cancer. Pathology was not available in four cases, leaving 435 for analysis. Overall, 165 (38%) of the patients underwent conservative surgery and 270 (62%) underwent mastectomy. In all, 33 (8%) of the patients had no residual invasive or noninvasive disease present after chemotherapy and 19 (4%) had residual DCIS only (Table 1

**Table 1:** Univariate analysis of factors predicting pCR in the breast following neoadjuvant chemotherapy

**Baseline factor**	**Total**	**pCR**		**Significance (*P*)**
Total	435	52	12.00%	

*ER*				
Positive	271	22	8.10%	<0.001
Negative	111	24	21.60%	
Unknown	53	6	11.30%	
				
*Menopausal status*				
Pre-meno	254	35	13.80%	0.4
Peri	39	5	12.80%	
Post	109	9	8.30%	
Hyst	33	3	9.10%	
				
*Clinical tumour size*				
T-1	5	2	40%	0.01
T-2	235	34	14.50%	
T-3	168	14	8.30%	
T-4	20	1	5.00%	
				
*Clinical nodes*				
Not palpable	240	31	12.90%	0.5
Palpable	195	21	10.80%	
				
*Grade*				
I	31	1	3.20%	<0.001
II	169	4	2.40%	
III	179	28	15.60%	
Not classifiable	56	19	33.90%	
				
*Chemotherapy*				
Anthracycline	359	50	13.90%	0.003
Nonanthracycline	76	2	2.60%	
Infusional	89	15	16.90%	0.1
Conventional	346	37	10.70%	

). Both of these groups were included in the pCR category, making the overall pCR rate 12%. A further five patients had a pCR in the breast, but had residual axillary lymph node involvement and therefore were not included in the pCR category. Of those women with ER-positive tumours, 66 were postmenopausal and six were aged over 55 and had undergone a hysterectomy. Three of these 72 women (4%) achieved a pCR following neoadjuvant chemotherapy.

On univariate analysis, factors associated with a pCR were ER status (*P*<0.001), primary tumour size by T-stage (*P*=0.01), tumour grade (*P*<0.001) and anthracycline *vs* nonanthracycline chemotherapy (*P*=0.003) (Table 1). Differences in pCR rate according to menopausal status and whether or not infusional chemotherapy was used were not statistically significant (Table 1). Tumour type was not routinely assessed on core biopsy; so this parameter was not included in the analysis.

A multivariate analysis was performed using the variables menopausal status, ER status, grade (I and II *vs* III) and anthracycline *vs* other chemotherapy. Oestrogen receptor status and grade were found to be highly correlated with 72% of ER-negative tumours also being grade 3, compared with 40% of ER-positive tumours. Owing to this correlation, ER status and tumour grade were not found to be independently significant prognostic indicators of pCR. However, when a statistical model was used where grade was excluded from the analysis, ER status was the main factor predicting pCR (*P*=0.001). After adjusting for this, the chemotherapy regimen has an additional marginal significance (*P*=0.02), with patients receiving anthracycline-based chemotherapy more likely to achieve a pCR than those receiving other regimens (Table 2

**Table 2:** Multivariate analysis of factors predicting pCR in the breast following neoadjuvant chemotherapy, using a model excluding grade from the analysis

**Factor**	**Odds ratio**	**95% CI**	**Significance (*P*)**
ER negative *vs* ER positive	3.08	(1.63, 5.81)	0.001
Anthracycline *vs* other chemotherapy	10.11	(1.37, 74.83)	0.02

).

### Survival

**Figure 1:**
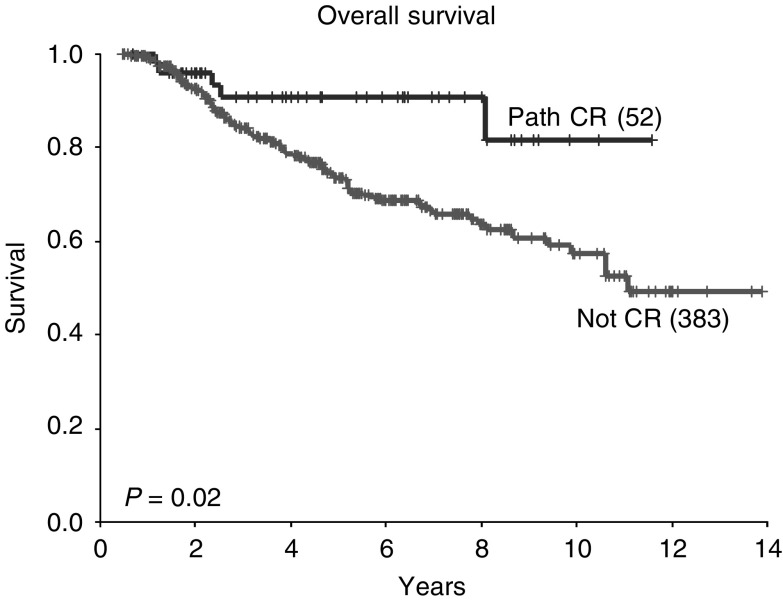
Overall survival according to pathological response (all patients).

**Figure 2:**
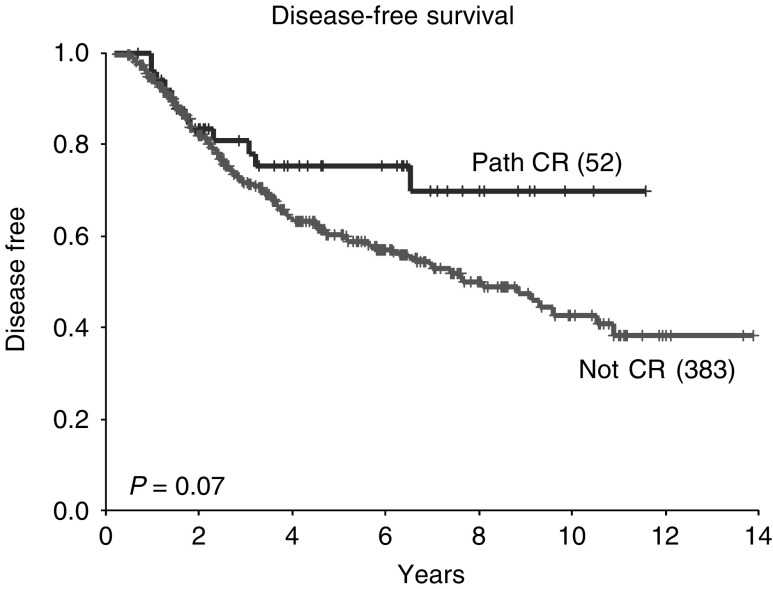
Disease-free survival according to pathological response (all patients).

**Figure 3:**
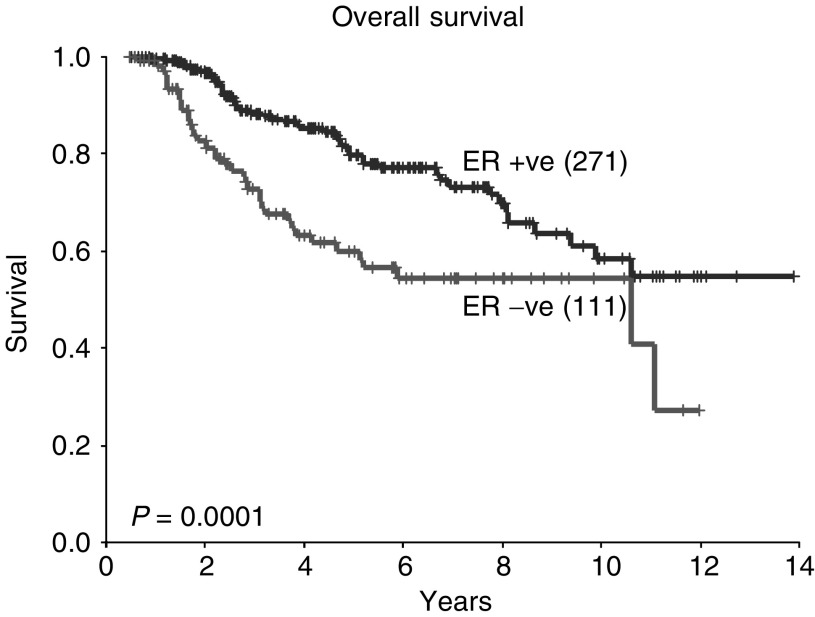
Overall survival according to ER status (all patients).

**Figure 4:**
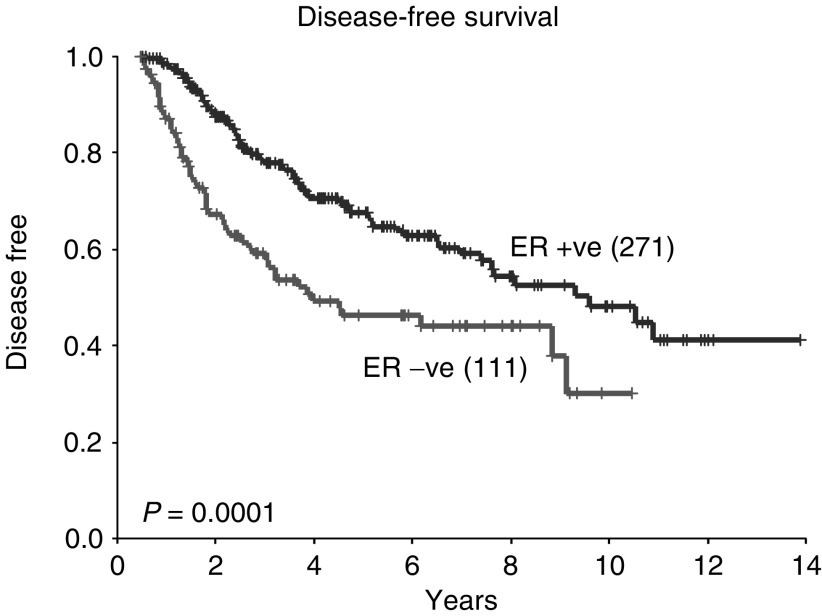
Disease-free survival according to ER status (all patients).

At a median follow-up of 53 months, the 5-year OS in all patients was 75% and the 5-year DFS 62%. A pCR following neoadjuvant chemotherapy was associated with improved survival (5-year OS 91 *vs* 73%; *P*=0.02, Figure 1

). There was a trend to improvement in DFS in patients achieving a pCR, but this did not reach statistically significant values (*P*=0.07, Figure 2

). Pathological complete response did not influence the rate of isolated local recurrences (*P*=0.5, data not shown). Negative ER status was associated with a worse OS, with a 5-year OS of 60% in ER-negative patients compared with 80% in ER-positive patients (*P*=0.0001, Figure 3

). Disease-free survival and isolated local recurrence rates were also inferior in patients with ER-negative tumours (*P*=0.0001 and 0.01, respectively, Figures 4

and 5

**Figure 5:**
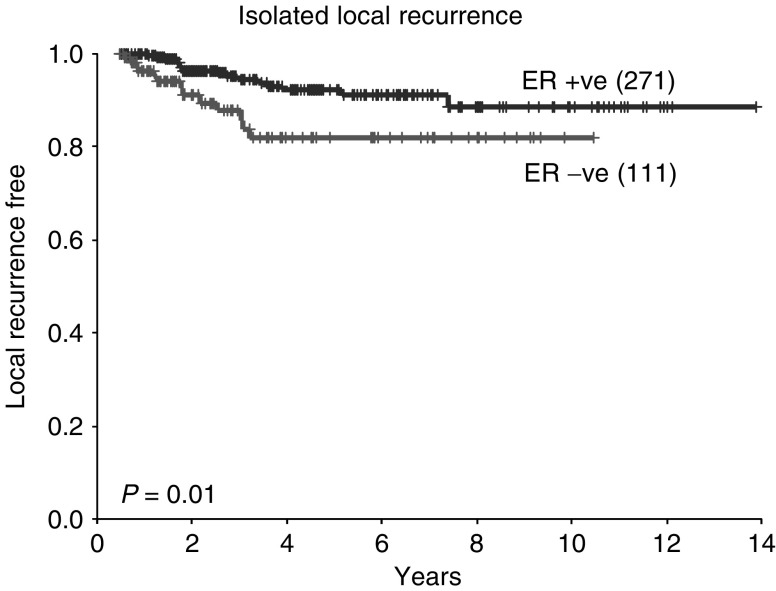
Isolated local recurrence rate according to ER status (all patients).

).

**Figure 6:**
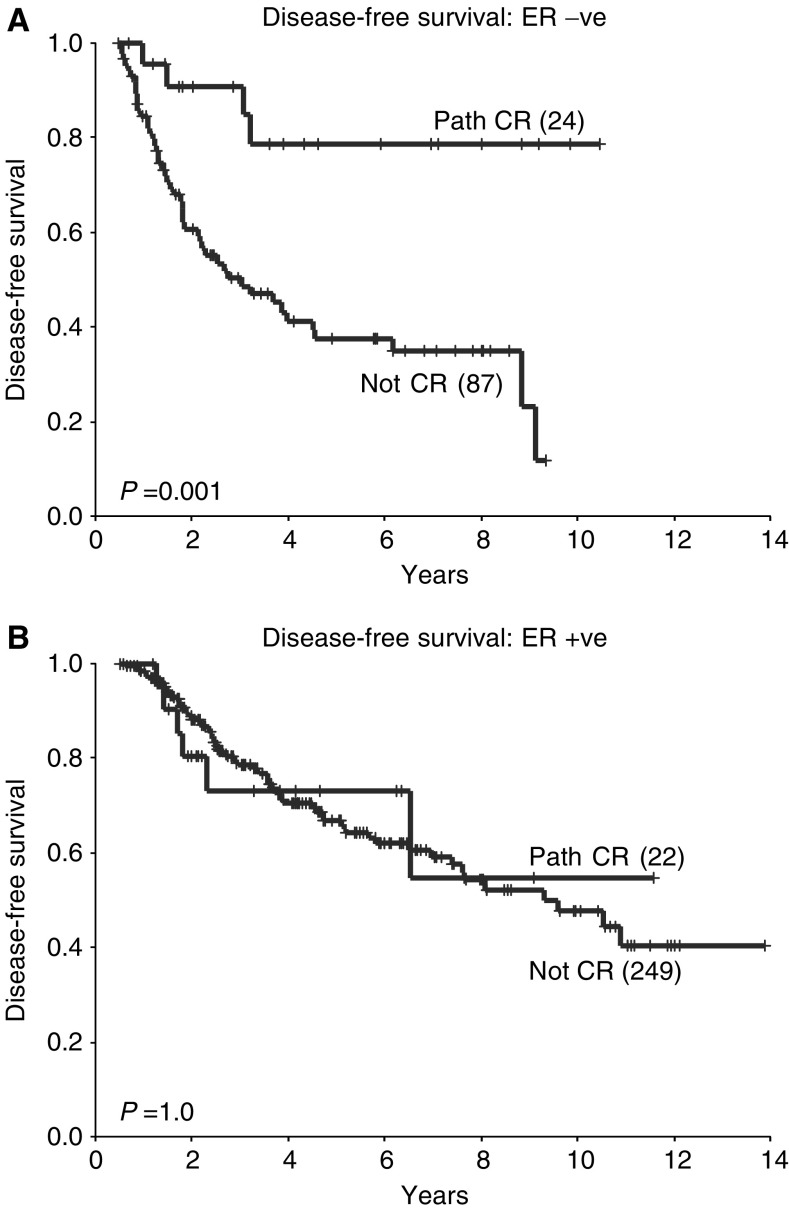
Disease-free survival according to pathological response in patients whose tumours are ER negative (**A**) and ER positive (**B**).

When only those patients with ER-negative tumours were examined, it was confirmed that those patients who achieved a pCR had an improved DFS and OS compared with those with residual disease (5-year DFS 73 *vs* 37%; *P*=0.001; 5-year OS 90 *vs* 52%; *P*=0.005, Figures 6A

and 7A

**Figure 7:**
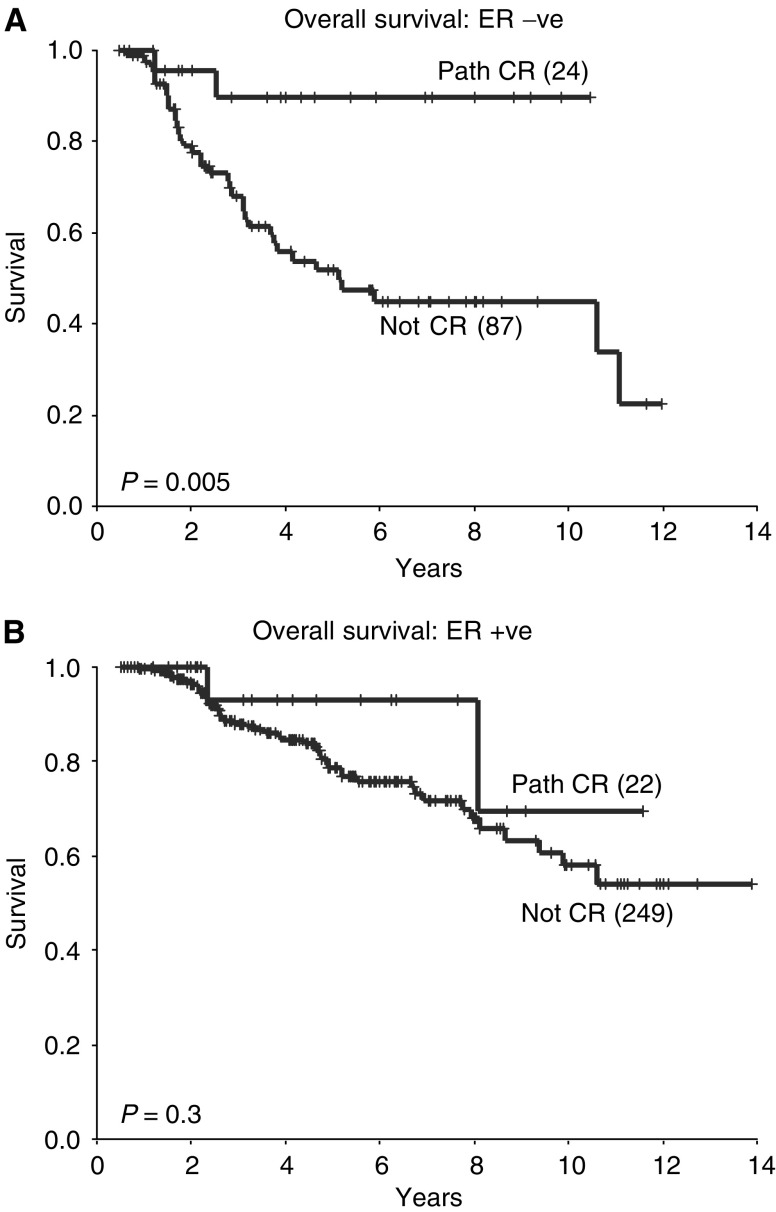
Overall survival according to pathological response in patients whose tumours are ER negative (**A**) and ER positive (**B**).

). However, this was not the case in patients with ER-positive tumours, where there were no significant differences in either DFS or OS between those who achieved a pCR and those who did not (5-year DFS 73 *vs* 67%; *P*=1.0; 5-year OS 93 *vs* 79%; *P*=0.3, Figures 6B and 7B). In addition, in those patients with ER-negative tumours who achieved a pCR, DFS and OS were at least comparable with those of patients with ER-positive tumours who achieved a pCR (5-year DFS 79 *vs* 73%; *P*=0.03) and those who did not (5-year DFS 79 *vs* 67%; *P*=0.2).

## DISCUSSION

This is a large single-centre analysis, with pathological response assessed by a single group of pathologists. It demonstrates that patients whose tumours are ER negative are more likely to achieve a pCR following neoadjuvant chemotherapy than those with ER-positive tumours. *In vitro* assays and clinical studies in the metastatic setting have shown that ER-negative breast cancers are more sensitive to chemotherapy than ER-positive tumours (Kaufmann *et al*, 1980; Rosner *et al*, 1989). Similarly, the Early Breast Cancer Trialists' Collaborative Group has shown that, with the use of adjuvant polychemotherapy, proportional reductions in recurrence are significantly greater for women with ER-poor tumours compared with those with ER-positive disease (Early Breast Cancer Trialists' Collaborative Group, 1998).

Previous studies have shown that ER-negative tumours are likely to have better clinical (as opposed to pathological) responses than ER-positive tumours (Bonadonna *et al*, 1990; Mauriac *et al*, 1991; MacGrogan *et al*, 1996). Colleoni *et al* (2000b) found a significantly higher clinical response rate in patients receiving neoadjuvant chemotherapy, in whose tumours ER and PgR were absent, compared to those where ER and/or PgR were expressed (82 *vs* 57%; *P*=0.03). However, the measurement of clinical responses is to an extent subjective, and the assessment of pathological response may be more robust and reproducible. Therefore, it is an important finding that, in the same study, at least on univariate analysis, pCR rates were also higher in the ER- and PgR-absent group (23 *vs* 7%; *P*=0.04; Colleoni *et al*, 2000b). Kuerer *et al* (1999) also found higher pCR rates in women with ER-negative locally advanced breast cancers treated with neoadjuvant chemotherapy (17 *vs* 3%; *P*<0.01, ER negative *vs* ER positive). In this study, the effects of ER status were found to be independent of initial tumour size on multivariate analysis, although ER status was not demonstrated to predict pCR independently of tumour grade.

In our study, on univariate analysis, ER-negative status was again shown to predict for pCR, with a pCR rate of 22% compared with 8% in ER-positive tumours (*P*<0.001). However, the strong correlation between ER status and tumour grade meant that neither could be demonstrated to be an independent prognostic indicator of pCR. Poor nuclear grade is recognised to predict pCR in the neoadjuvant setting (Fisher *et al*, 2002). There are, however, practical issues in getting reproducible measurements of tumour grade from the small amount of tissue acquired at core biopsy, and previous studies have shown that tumour grade assessed on core biopsy may not be representative of the final surgical specimen (Connor *et al*, 2002; Harris *et al*, 2003). In contrast, ER status assessed on core biopsy shows good concordance with the surgical specimen (Jacobs *et al*, 1998; Connor *et al*, 2002) and, when a statistical model excluding grade was used in this analysis, ER status was the main factor predicting pCR.

This is an important consideration when interpreting trials of neoadjuvant chemotherapy. The ER status of the trial population should be carefully examined. High pCR rates in unrandomised trials and differences in pCR rates between treatments in randomised trials could be explained by high proportions of ER-negative patients and imbalances in ER status between treatment arms, respectively.

It should be recognised that the cutoff used to define ER-negative status differs between studies in this area. In our analysis, in common with historical practice, patients were classified as ER positive (⩾10 fmol mg^−1^ cytosol protein or ⩾10% of positive cells) or ER negative (⩽10 fmol mg^−1^ cytosol protein or <10% of positive cells). However, the study published by Colleoni *et al* (2000b) defined an ER-absent group as one with 0% cells positive and ER positive when ⩾1% of cells stained positive. Similarly, the IBCSG has previously defined three groups on the basis of ER content: ER absent, ER low (1–9 fmol mg^−1^ cytosol protein) and ER positive (⩾10 fmol mg^−1^ cytosol) (Colleoni *et al*, 2000a). This was based on data showing a better prognosis for tumours with as few as 1% of cells staining positive when treated with tamoxifen (Harvey *et al*, 1999).

The survival data presented in this analysis concur with previous studies in that overall patients who are ER positive and those who achieve a pCR following neoadjuvant chemotherapy have a better prognosis. However, an important additional finding is that, when patients with ER-negative tumours achieve a pCR, their survival is comparable with that of ER-positive patients. This is despite the fact that overall patients with ER-negative tumours have a worse prognosis than those with ER-positive tumours. Therefore, in patients with ER-negative tumours, the degree of pathological response following neoadjuvant chemotherapy may provide important prognostic information. This may have practical implications for subsequent treatment and follow-up.

A second novel finding was that, although patients with ER-negative tumours achieving a pCR had an improved prognosis, this was not the case in patients who had ER-positive tumours. The latter had similar DFS and OS regardless of pathological response. This represents a potentially important finding which, if corroborated, would imply that the surrogate outcome measure pCR, used in many neoadjuvant chemotherapy trials, may not be a valid predictor of final outcome in patients with ER-positive tumours.

As with all retrospective studies, there are limitations to this analysis and the results should be interpreted with caution. Only small numbers of patients are included and in particular very few patients with ER-positive tumours achieved a pCR meaning that the survival data are difficult to interpret. Although pathological assessment was undertaken at a single institution with protocols that adhered to National guidelines, there is likely to have been variability over the years in ascertainment of histological grade, ER status and the number of tumour blocks examined. In addition, ER status was not known for 53 of the 435 patients, and a population of patients who did not undergo surgery was excluded from the analysis, introducing further bias.

If the results of this small retrospective analysis are replicated in future prospective studies, there may be important implications not only in terms of prediction of response and survival, but also in terms of choice of neoadjuvant therapy. The relatively low pCR rate following neoadjuvant chemotherapy in those patients with ER-positive tumours might suggest that alternative approaches such as primary surgery or hormonal therapy may be preferable. In particular, the low pCR rate in postmenopausal women with ER-positive tumours may mean that primary aromatase inhibitor treatment is an attractive option.
